# An Excellent Monitoring System for Surface Ubiquitination-Induced Internalization in Mammals

**DOI:** 10.1371/journal.pone.0001490

**Published:** 2008-01-30

**Authors:** Eiji Goto, Mari Mito-Yoshida, Mika Uematsu, Masami Aoki, Yohei Matsuki, Mari Ohmura-Hoshino, Hak Hotta, Makoto Miyagishi, Satoshi Ishido

**Affiliations:** 1 Laboratory for Infectious Immunity, RIKEN Research Center for Allergy and Immunology, Yokohama, Kanagawa, Japan; 2 Department of Pathology and Microbiology, Kobe University Graduate School of Medicine, Kobe, Hyogo, Japan; 3 21st Century COE Program, School of Medicine, The University of Tokyo, Tokyo, Japan; University of Geneva, Switzerland

## Abstract

**Background:**

At present, it is difficult to visualize the internalization of surface receptors induced by ubiquitination that is taken place at the plasma membrane in mammals. This problem makes it difficult to reveal molecular basis for ubiquitination-mediated internalization in mammals.

**Methodology/Principle Findings:**

In order to overcome it, we have generated T-REx-c-MIR, a novel mammalian Tet-on B cell line using a constitutively active E3 ubiquitin ligase, c-MIR, and its artificial target molecule. By applying the surface biotinylation method to T-REx-c-MIR, we succeeded to monitor the fate of surface target molecules after initiation of ubiquitination process by doxycycline (Dox)-induced c-MIR expression. Target molecules that pre-existed at the plasma membrane before induction of c-MIR expression were oligo-ubiquitinated and degraded by Dox-induced c-MIR expression. Dox-induced c-MIR expression initiated rapid internalization of surface target molecules, and blockage of the internalization induced the accumulation of the surface target molecules that were newly ubiquitinated by c-MIR. Inhibition of the surface ubiquitination by down-regulating ubiquitin conjugating enzyme E2 impaired the internalization of target molecules. Finally, a complex of c-MIR and target molecule was detected at the plasma membrane.

**Conclusions/Significances:**

These results demonstrate that in T-REx-c-MIR, surface target molecule is ubiquitinated at the plasma membrane and followed by being internalized from the plasma membrane. Thus, T-REx-c-MIR is a useful experimental tool to analyze how surface ubiquitination regulates internalization in mammals.

## Introduction

Ubiquitination is a well-known post-transcriptional modification in eukaryote. Ubiquitination plays important roles in gene transcription, membrane traffic, and signal transduction [1]. During ubiquitination, a small molecule named ubiquitin, whose MW is 8 kD, is covalently conjugated to the target substrate through two different modes: mono-ubiquitination and poly-ubiquitination. Conjugation with one ubiquitin molecule and with tandem repeated ubiquitin molecules is defined as mono- and poly-ubiquitination, respectively. Among the cases of poly-ubiquitination, conjugation with a few ubiquitin molecules is called oligo-ubiquitination. Ubiquitination is sequentially achieved by the cooperation of three enzymes: ubiquitin activating enzyme (E1), ubiquitin conjugating enzyme (E2), and ubiquitin ligase (E3). Among these enzymes, E3 determines its specific substrate for ubiquitination by binding to the molecule to be ubiquitinated [1].

Classically, ubiquitination is recognized as a signal for degradation at the proteasome [2]. In the case of incorrectly folded proteins, these undesired proteins undergo K48-linked chain ubiquitination and are degraded at the proteasome [2]. These systems are required to maintain the quality of functional proteins. Several receptors that induce signals required for development and homeostasis are ubiquitinated. Unlike incorrectly folded proteins, these ubiquitinated receptors are degraded in the lysosome [3], [4]. This ubiquitination-mediated lysosomal degradation functions as a negative feedback signal and is required to maintain homeostasis [5]. Indeed, several mutations that disrupt the ubiquitination activity of c-Cbl, an E3 for the EGF receptor, have been found in cancer [6]. Cancer cells carrying a mutation in the RING domain of c-Cbl cannot down-regulate signaling from the EGF receptor. Also, the mutant form of growth factor receptor that cannot associate with a responsible E3 induces sustained signals and promotes hematopoietic malignancy (e.g., leukemia) [7], [8]. Thus, the investigation of ubiquitination-mediated membrane trafficking is important to understand how homeostasis is regulated in vivo.

Among the many steps involved in the ubiquitination-mediated membrane trafficking, internalization from the plasma membrane is the first and most critical step for the desensitization of signaling derived from several receptors [9]. Using yeast as an experimental model, the molecular mechanisms underlying ubiquitination-mediated membrane trafficking were intensively investigated [3], [9], [10]. It has been clearly demonstrated that surface ubiquitination initiates the internalization of plasma membrane proteins including receptors for G-proteins, by analyzing several mutants of yeast [3]. However in mammals, it is difficult to analyze the relationship between surface ubiquitination and internalization of target surface molecules as there are no suitable experimental models in mammals.

In order to overcome this problem, we have established a novel Tet-on B cell line named T-REx-c-MIR by employing a constitutively active ubiquitin ligase, c-MIR [11]–[14]. As we have reported before, doxycycline (Dox)-induced c-MIR expression leads to ubiquitination and internalization of target molecules (e.g., MHC class II (MHC II) and B7-2). By applying the surface biotinylation method to this cell line, we succeeded in clearly monitoring the fate of surface target molecules after initiation of ubiquitination by Dox-mediated c-MIR expression. Blockage of the internalization with pharmacological reagents induced the accumulation of target surface molecules that were newly ubiquitinated by c-MIR. Inhibition of surface ubiquitination by down-regulating E2 impaired internalization of the target surface molecule. In addition, a complex of c-MIR and target molecule was detected on the plasma membrane. These results demonstrate that in T-REx-c-MIR, surface target molecule is ubiquitinated at the plasma membrane and followed by being internalized from the plasma membrane. Thus, T-REx-c-MIR is a useful experimental tool to analyze how surface ubiquitination regulates internalization in mammals.

## Results

### Generation of T-REx-c-MIR

**Figure 1:**
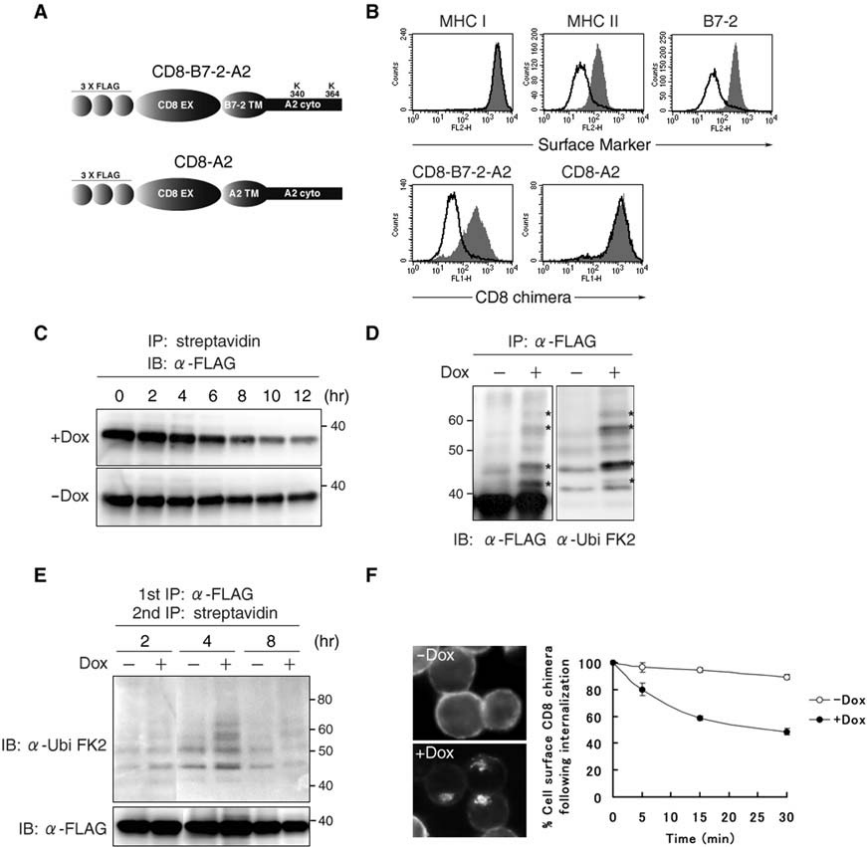
Generation of T-REx-c-MIR. (A) Schematic representation of the structure of CD8 chimeras used in this experiment. Extracellular domain (EX), transmembrane domain (TM), and cytoplasmic tail (cyto) are indicated. (B) After being incubated with and without Dox for 24 hr, the expression level of indicated surface molecules on T-REx-c-MIR was examined by FACS. Data from the cells incubated with Dox (open histograms), and the cells incubated without Dox (shaded histograms) are shown. Data are representative of two independent experiments. (C) Surface molecules of T-REx-c-MIR were biotinylated first. After biotinylated T-REx-c-MIR was incubated with or without Dox for the indicated periods of times, biotinylated proteins were purified with streptavidin-agarose and analyzed with anti-FLAG Ab. Upper and lower panels showed the results from the cells incubated with and without Dox, respectively. Data are representative of two independent experiments. (D) Whole cell lysate extracted from T-REx-c-MIR that was cultivated with or without Dox for 6 hr was incubated with anti-FLAG Ab. Precipitated samples were probed with anti-FLAG Ab (left) or anti-ubiquitin Ab (right). Bands corresponding to the ubiquitinated CD8-B7-2-A2 is are marked by an asterisk (*) as shown. (E) Surface molecules of T-REx-c-MIR were biotinylated as in C. After biotinylated T-REx-c-MIR was incubated with (+) or without (−) Dox for the indicated periods of time, biotinylated proteins were sequentially purified with anti-FLAG Ab and streptavidin-agarose. Each sample was probed with anti-ubiquitin Ab (upper) and anti-FLAG Ab (lower). Data are representative of two independent experiments. (F) T-REx-c-MIR was incubated with Dox for 6 hr and cultivated in the presence of FITC-conjugated anti-CD8 Ab for the last 10 min. Internalized CD8-B7-2-A2 was observed with a fluorescence microscope (left panel). For the quantitative analysis of internalization, surface CD8-B7-2-A2 of T-REx-c-MIR was labeled with anti-CD8 Ab after being incubated with (+) or without (−) Dox for 6 hr. After cultivation at 37°C for the indicated times, the expression of remaining surface CD8-B7-2-A2 was examined by staining with PE-conjugated goat anti-mouse IgG. At each point, the percentage of remaining CD8-B7-2-A2 was calculated relative to the value of labeled CD8-B7-2-A2 at 0 min (right panel).

In order to monitor the surface events initiated by ubiquitination in mammals, we have generated a novel experimental tool, Tet-on B cell line, and named it T-REx-c-MIR. Using this system, we were able to observe specific down-regulation of target molecules by doxycycline (Dox)-induced c-MIR expression. c-MIR was expressed within 2 hr after adding Dox, and this was followed by down-regulation of the surface expression of MHC II and B7-2, which are substrates for c-MIR [11], [13] (data not shown, Figure 1B). In contrast, MHC class I (MHC I), which is not a substrate for c-MIR, was not down-regulated (Figure 1B). We examined whether the ubiquitination of endogenous B7-2 molecules is detectable in T-REx-c-MIR. Unfortunately, we could not detect the ubiquitinated form of B7-2 unless these cells were treated with bafilomycin A1 (Figure S1), which has been shown to be a potent inhibitor for c-MIR-induced degradation in our previous work [11]. Therefore, CD8-B7-2-A2, a FLAG-tagged CD8 chimeric target molecule, was expressed in this system to improve visualization of the ubiquitination status of target molecules (Figure 1A). CD8-B7-2-A2 consists of an amino-terminal FLAG-tagged CD8 extracellular domain, a B7-2 transmembrane domain, and a cytoplasmic tail of MHC I, and can be efficiently purified with anti-FLAG M2 beads (Figure 1A). As shown in Figure 1B, CD8-B7-2-A2, but not CD8-A2, which consists of an HLA-A2 transmembrane domain instead of the B7-2 transmembrane domain, was efficiently down-regulated, demonstrating that CD8-B7-2-A2 can be utilized as a substrate for c-MIR in this system.

First, we examined whether surface CD8-B7-2-A2 is degraded by Dox-induced c-MIR. Surface molecules of T-REx-c-MIR were biotinylated with a membrane-impermeable reagent first, and biotinylated T-REx-c-MIR was incubated at 37°C in the presence of Dox for the indicated times. At the end of each incubation, biotinylated proteins including CD8-B7-2-A2 were purified with streptavidin beads and subjected to western blot analysis with anti-FLAG M2 antibody (Ab). As shown in Figure 1C, after induction for 6–8 hr, the amount of surface CD8-B7-2-A2 was significantly decreased compared with that without induction.

Next, the status of ubiquitination of CD8-B7-2-A2 was analyzed by purification from whole cell lysate. To prevent contamination with CD8-B7-2-A2-associated molecules, CD8-B7-2-A2 was purified from protein samples boiled in 1% SDS-containing RIPA buffer. Purified CD8-B7-2-A2 was analyzed with anti-FLAG and anti-ubiquitin Abs. As marked with asterisks in Figure 1D, additional bands recognizable with both anti-FLAG and anti-ubiquitin Abs appeared when Dox was added, confirming that CD8-B7-2-A2 itself was ubiquitinated by c-MIR in T-REx-c-MIR. Furthermore, we investigated whether surface CD8-B7-2-A2 is ubiquitinated by Dox-induced c-MIR. After surface molecules including CD8-B7-2-A2 on T-REx-c-MIR were biotinylated with a membrane-impermeable reagent, c-MIR was expressed by adding Dox in biotinylated T-REx-c-MIR. After induction of c-MIR expression, CD8-B7-2-A2 that pre-exists at the plasma membrane before the process of ubiquitination starts was purified by two sequential steps at each indicated time point (see Materials and methods). Purified CD8-B7-2-A2 was subjected to western blot analysis with anti-FLAG and anti-ubiquitin Abs. As shown in Figure 1E, surface CD8-B7-2-A2 was clearly ubiquitinated after incubation with Dox for 4 hr, and ubiquitinated CD8-B7-2-A2 was degraded after incubation for 8 hr (Figure 1E). Without the procedure of surface biotinylation, we could not observe any signals (data not shown).

Lastly, we examined whether CD8-B7-2-A2 is internalized from plasma membrane. After induction with Dox for 6 hr, surface CD8-B7-2-A2 was labeled with FITC-conjugated anti-CD8 Ab, and the trafficking of labeled CD8-B7-2-A2 was examined by fluorescence microscopy. As shown in Figure 1F, labeled CD8-B7-2-A2 molecules were efficiently internalized by adding Dox. These data were confirmed by using FACS-based analysis (Figure 1F). Taken together, these data demonstrate that T-REx-c-MIR is a useful experimental tool to analyze how ubiquitination contributes to the internalization and degradation of target surface molecules.

### Accumulation of ubiquitinated surface CD8 chimera by inhibiting internalization

**Figure 2:**
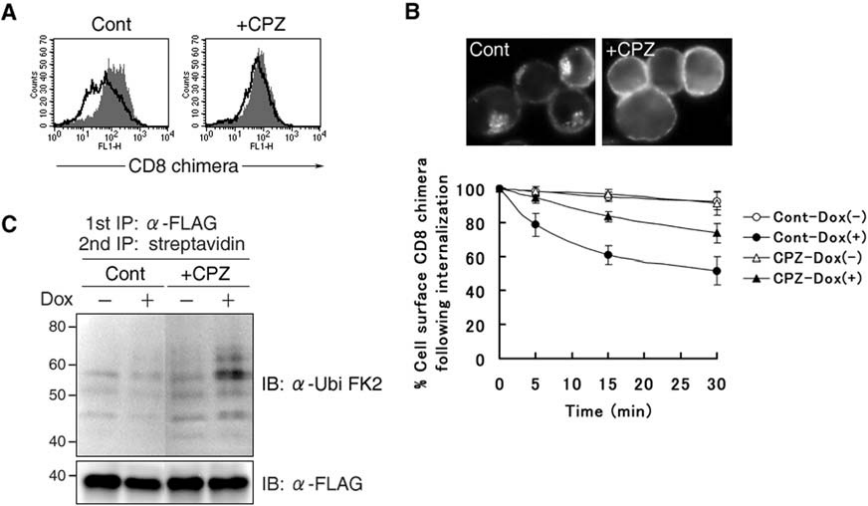
Accumulation of ubiquitinated surface CD8 chimera by inhibiting internalization. (A) After being incubated with or without Dox for 3 hr, T-REx-c-MIR was further cultivated for 5 hr with chlorpromazine (10 µg/ml) (+CPZ) or water (Cont) in the presence or absence of Dox. At the end of incubation, the expression of surface CD8-B7-2-A2 on T-REx-c-MIR was examined by FACS. Data from cells incubated with Dox (open histograms) and cells incubated without Dox (shaded histograms) are shown. Data are representative of two independent experiments. (B) T-REx-c-MIR was treated as in A. Treated T-REx-c-MIR was incubated in the presence of FITC-conjugated CD8 Ab at 37°C for 10 min, and internalized CD8-B7-2-A2 was examined by fluorescence microscopy. For the quantitative analysis of internalization, surface CD8-B7-2-A2 of T-REx-c-MIR treated as above was labeled with anti-CD8 Ab, and cultivated at 37°C for the indicated times. The expression of remaining surface CD8-B7-2-A2 was examined by staining with PE-conjugated goat anti-mouse IgG. At each point, the percentage of remaining CD8-B7-2-A2 was calculated as in Figure 1F. (C) Surface molecules of T-REx-c-MIR were biotinylated first as in Figure 1C. After biotinylated T-REx-c-MIR was incubated with (+) or without (−) Dox for 3 hr, biotinylated T-REx-c-MIR was further cultivated with chlorpromazine (10 µg/ml) (+CPZ) or water (Cont) for 5 hr in the presence (+) or absence (−) of Dox. After cultivation, biotinylated CD8-B7-2-A2 was sequentially purified with anti-FLAG Ab and streptavidin-agarose. Each sample was probed with anti-ubiquitin Ab (upper panel) or anti-FLAG Ab (lower panel). Data are representative of two independent experiments.

The experiments performed above revealed that surface CD8-B7-2-A2 is indeed ubiquitinated by the induced c-MIR expression, but did not show that the ubiquitination of surface CD8-B7-2-A2 takes place at the plasma membrane. Therefore, we examined whether ubiquitinated surface CD8 chimera is accumulated by inhibiting internalization. To this end, we examined the ability of several pharmacological reagents to inhibit internalization induced by c-MIR. Among the reagents examined, chlorpromazine and dansylcadaverine, which are inhibitors of clathrin-dependent internalization, significantly inhibited the down-regulation of CD8-B7-2-A2 without inhibiting c-MIR expression induced by Dox (data not shown, Figure 2A). Consistent with this, the internalization of surface CD8 molecules was inhibited by these reagents (Figure 2B). These results suggest that c-MIR induces the internalization of surface CD8-B7-2-A2 in a clathrin-dependent manner.

Based on these results, we employed chlorpromazine for the following experiments. After surface molecules of T-REx-c-MIR were biotinylated, c-MIR was expressed by adding Dox in the presence or absence of chlorpromazine. After incubation of biotinylated T-REx-c-MIR with Dox for 8 hr, surface biotinylated CD8-B7-2-A2 was purified and analyzed as performed in Figure 1E. As shown in Figure 2C, in the presence of chlorpromazine (+CPZ), ubiquitinated form of biotinylated CD8-B7-A2 was clearly detected even by adding Dox (+), demonstrating that the surface CD8-B7-2-A2 that was newly ubiquitinated by c-MIR was accumulated by blockage of internalization. These results demonstrate that the ubiquitination of CD8-B7-2-A2 takes place at the plasma membrane in T-REx-c-MIR.

### Lysine-less target molecules are neither ubiquitinated nor internalized in T-REx-c-MIR

**Figure 3:**
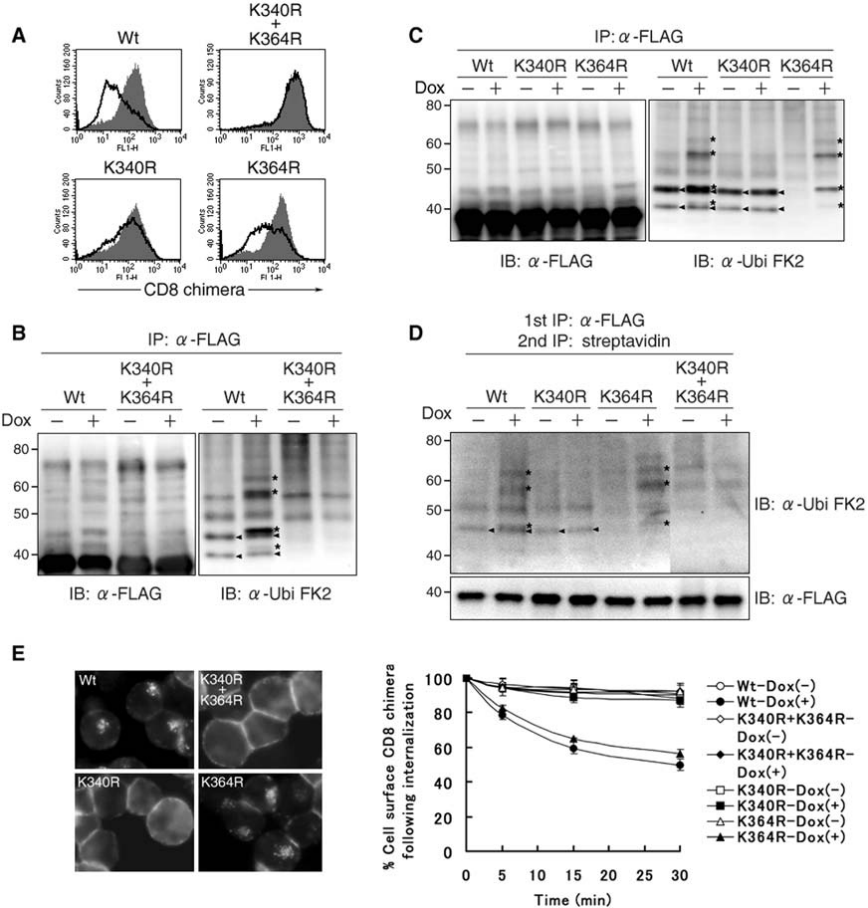
Lysine-less target molecules are neither ubiquitinated nor internalized in T-REx-c-MIR. (A) After being incubated with and without Dox for 8 hr, the expression of indicated surface molecules on T-REx-c-MIR was examined by FACS. Data from cells incubated with Dox (open histograms) and cells incubated without Dox (shaded histograms) are shown. Data are representative of two independent experiments. (B) (C) Whole cell lysate extracted from indicated CD8-chimera-expressing T-REx-c-MIR that was cultivated with or without Dox for 6 hr was incubated with anti-FLAG Ab. Precipitated samples were probed with anti-FLAG Ab (left) or anti-ubiquitin Ab (right). Bands corresponding to newly and constitutively ubiquitinated CD8-B7-2-A2 are marked with an asterisk (*) and small triangles, respectively (shown in right panels). (D) Surface molecules of indicated CD8-chimera-expressing T-REx-c-MIR were biotinylated first as in Figure 1C. After each biotinylated T-REx-c-MIR was incubated with (+) or without (−) Dox for 4 hr, biotinylated proteins were sequentially purified with anti-FLAG Ab and streptavidin-agarose. Each sample was probed with anti-ubiquitin Ab or anti-FLAG Ab. Data are representative of two independent experiments. Bands corresponding to newly and constitutively ubiquitinated surface CD8-B7-2-A2 are marked with an asterisk (*) and small triangles, respectively. (E) Indicated CD8-chimera-expressing T-REx-c-MIR was incubated with Dox for 6 hr and cultivated in the presence of FITC-conjugated anti-CD8 Ab for the last 10 min. Internalized CD8 chimera was observed by fluorescence microscopy (left panel). For the quantitative analysis of internalization, each T-REx-c-MIR was incubated with (+) or without (−) Dox for 6 hr, and surface CD8 chimera of T-REx-c-MIR was labeled with anti-CD8 Ab. After each labeled T-REx-c-MIR was cultivated at 37°C for the indicated times, the percentage of remaining CD8-B7-2-A2 was calculated as in Figure 1F (right panel).

We examined whether non-ubiquitinated surface CD8-B7-2-A2 is less internalized in T-REx-c-MIR. Since CD8-B7-2-A2 has two lysine residues, K340 and K364, at its cytoplasmic tail, which are candidates for the ubiquitination site, these candidate sites were mutated to arginine simultaneously or one by one. Each CD8-B7-2-A2 mutant was stably expressed in T-REx-c-MIR and assayed. Eight hours after induction by Dox, the surface expression of each CD8-B7-2-A2 mutant was examined by FACS analysis. As shown in Figure 3A, the CD8-B7-2-A2 mutant whose two lysine residues were simultaneously mutated to arginine, named CD8-B7-2-A2^K340R, K364R^, was not down-regulated at all. Among the two responsible lysine residues, K340 had a greater effect than K364 in down-regulating the target molecule (Figure 3A).

To investigate whether the inhibition of down-regulation is due to less ubiquitination, the status of ubiquitination of CD8-B7-2-A2 mutants was examined, as performed in Figure 1D. As shown in Figure 3B, the status of ubiquitination of CD8-B7-2-A2^K340R, K364R^, which was purified from whole cell lysate, was not changed by Dox-induced c-MIR expression. Furthermore, the status of ubiquitination of CD8-B7-2-A2^K340R, K364R^ was different from that of CD8-B7-2-A2 even without adding Dox; the two bands marked with small triangles in the right panel of Figure 3B and C disappeared in CD8-B7-2-A2^K340R, K364R^. Those bands also disappeared in CD8-B7-2-A2^K364R^, in which K364 of CD8-B7-2-A2 was mutated to arginine, demonstrating that the ubiquitination of K364 takes place in the steady state (Figure 3C). On the other hand, the bands that appeared due to Dox-induced c-MIR (marked with * in Figure 3B and C) disappeared in CD8-B7-2-A2^K340R^, in which K340 of CD8-B7-2-A2 was mutated to arginine (Figure 3C). This was also the case in the surface CD8-B7-2-A2 mutants (Figure 3D). Together, the results indicate that Dox-induced c-MIR promotes internalization of CD8-B7-2-A2 most likely through ubiquitination at K340.

To confirm this, we examined the internalization rate of each mutant. As shown in Figure 3E, the internalization rate of CD8-B7-2-A2^K340R, K364R^ was the lowest among the mutants examined. Consistent with the results shown in Figure 3A, the internalization rate of CD8-B7-2-A2^K340R^ was lower than that of CD8-B7-2-A2^K364R^. Taken together, these results strongly suggest that ubiquitination at K340 mainly contributes to c-MIR-mediated internalization.

### Inhibition of surface ubiquitination weakens internalization

The results obtained by analysis with mutant target molecules support the idea that surface ubiquitination functions as an internalization signal in T-REx-c-MIR, but must be interpreted with care. Lysine residues might be modulated by a conjugation system that has not yet been identified to this day. Therefore, we examined the effect of inhibiting surface ubiquitination on the internalization rate of CD8-B7-2-A2. It was demonstrated that UbcH5b and c are responsible ubiquitin conjugating enzymes for MIR1, a viral ubiquitin ligase that belongs to the same family as c-MIR [15]. Also, we showed that the RINGv domain of c-MIR can function as an E3 catalytic domain of MIR1 [11]. Therefore, we examined the possibility that c-MIR also utilizes UbcH5b and c as ubiquitin conjugating enzymes, using the retroviral transduction method.

**Figure 4:**
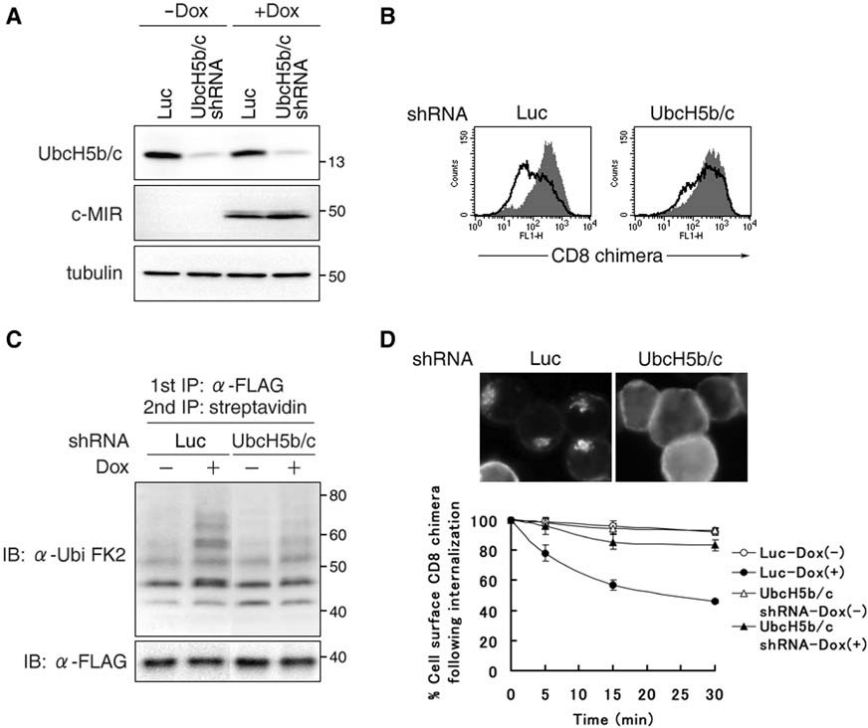
Inhibition of surface ubiquitination weakens internalization. (A) Control shRNA (Luc) or shRNA for UbcH5b and c (UbcH5b/c) was retrovirally transduced into T-REx-c-MIR. Whole cell lysate from each indicated T-REx-c-MIR was analyzed with anti-UbcH5, V5 (for c-MIR), and tubulin Abs 6 hr after incubation with or without Dox. Data are representative of two independent experiments. (B) Indicated T-REx-c-MIR was incubated with or without Dox for 8 hr and the expression of CD8-B7-2-A2 was analyzed by FACS. Data are representative of two independent experiments. (C) Surface molecules of T-REx-c-MIR treated with indicated shRNA were biotinylated first as in Figure 1C. After biotinylated T-REx-c-MIR was incubated with (+) or without (−) Dox for 4 hr, biotinylated CD8-B7-2-A2 was sequentially purified with anti-FLAG Ab and streptavidin-agarose. Each sample was probed with anti-ubiquitin Ab or anti-FLAG Ab. Data are representative of two independent experiments. (D) Indicated T-REx-c-MIR was incubated with Dox for 6 hr and cultivated in the presence of FITC-conjugated anti-CD8 Ab for the last 10 min. Internalized CD8 chimera was observed by fluorescence microscopy (upper panel). For the quantitative analysis of internalization, indicated T-REx-c-MIR was incubated with (+) or without (−) Dox for 6 hr, and surface CD8-B7-2-A2 of T-REx-c-MIR was labeled with anti-CD8 Ab. After cultivation at 37°C for the indicated times, the percentage of remaining CD8-B7-2-A2 was calculated as in Figure 1F (lower panel).

T-REx-c-MIR, whose UbcH5b/c expression was knocked down, was generated by infection with a retrovirus that expresses shRNA against both UbcH5b and c (Figure 4A). c-MIR was expressed by adding Dox in knocked down T-REx-c-MIR, and the surface expression of CD8-B7-2-A2 was examined. As we expected, the down-regulation of CD8-B7-2-A2 was significantly inhibited in knocked down T-REx-c-MIR compared with T-REx-c-MIR infected with control shRNA-expressing retrovirus (Figure 4A). In both cells, the expression of c-MIR was equivalent (Figure 4A). Therefore, we employed shRNA for UbcH5b/c to inhibit c-MIR-mediated ubiquitination. As shown in Figure 4C, D, surface CD8-B7-2-A2 was less ubiquitinated and was less internalized in knocked down T-REx-c-MIR compared with control cells. These results clearly demonstrate that surface ubiquitination is necessary for internalization of surface CD8 chimera.

### Complex formation between CD8 chimera target and c-MIR at plasma membrane

**Figure 5:**
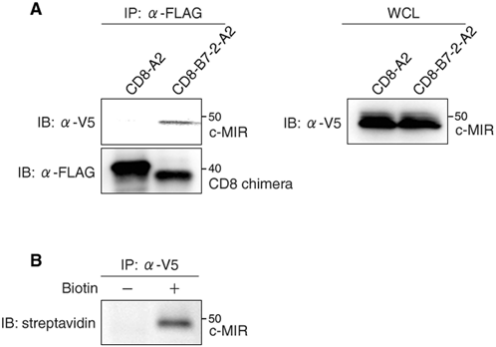
c-MIR binds to CD8 chimera target at plasma membrane. (A) Indicated CD8-chimera-expressing T-REx-c-MIR was incubated with Dox for 4 hr, and surface molecules of T-REx-c-MIR were biotinylated as in Figure 1C. Protein complex including biotinylated CD8 chimera was sequentially purified with anti-FLAG Ab and streptavidin-agarose. Each sample was probed with anti-V5 Ab and anti-FLAG Ab (left panel). Whole cell lysate from each T-REx-c-MIR was analyzed with anti-V5 Ab (right panel). Data are representative of two independent experiments. (B) T-REx-c-MIR was incubated with Dox for 4 hr, and surface molecules of T-REx-c-MIR were biotinylated. c-MIR proteins were purified with anti-V5 Ab and analyzed with HRP-conjugated streptavidin. Data are representative of two independent experiments.

Lastly, we examined whether CD8-B7-2-A2 forms a complex with c-MIR at the plasma membrane. As negative control, CD8-A2 was employed because it does not bind to c-MIR and therefore is not ubiquitinated by c-MIR [11]. Surface molecules of T-REx-c-MIR, which constitutively express CD8-B7-2-A2 or CD8-A2, were biotinylated after c-MIR was expressed by adding Dox, and the protein complex including biotinylated CD8-B7-2-A2 or CD8-A2 was purified with streptavidin beads and anti-FLAG M2 beads. Purified protein complex was subjected to western blot analysis with anti-V5 and anti-FLAG Abs to detect c-MIR and CD8 chimeras, respectively. As shown in Figure 5A, c-MIR was detected in the protein complex containing surface CD8-B7-2-A2, but not in the protein complex containing surface CD8-A2. Furthermore, we examined whether c-MIR is present at the plasma membrane. After c-MIR was expressed by adding Dox, surface molecules of T-REx-c-MIR were biotinylated. Expressed c-MIR was purified with anti-V5 Ab and analyzed with HRP-conjugated streptavidin. As shown in Figure 5B, biotinylated c-MIR was clearly detected. These results demonstrate that c-MIR associates with CD8-B7-2-A2 at the plasma membrane.

## Discussion

Here, we reported a useful experimental tool named as T-REx-c-MIR for monitoring the internalization induced by surface ubiquitination. This construction was achieved by employing a constitutively active E3 ubiquitin ligase, c-MIR. Given that impaired ubiquitin-mediated regulation of growth factor receptors is suggested to cause several diseases including malignancies [6], the molecular machinery of ubiquitin-mediated internalization, which will be revealed by using T-REx-c-MIR, will provide new insights into strategies for treatment of human diseases.

In mammals, it is difficult to reveal molecular basis for ubiquitination-mediated internalization, because it has been difficult to obtain the proof that surface ubiquitination functions an internalization signal for the following reasons. One is the presence of possible redundant pathways for the internalization of surface molecules in mammals. For instance, in the case of the EGF receptor (EGFR) that is under intensive investigation, two machineries for internalization have been proposed [3]. The first one is the c-Cbl-mediated ubiquitination of EGFR, where the binding of EGF to EGFR induces auto-phosphorylation of several cytoplasmic tyrosine residues of EGFR and recruits c-Cbl, a ubiquitin ligase, through the SH-2 domain of c-Cbl, thereby ubiquitinating EGFR at multiple cytoplasmic lysine residues and inducing internalization. The second one is the CIN-85-mediated internalization, where, upon receptor activation, EGFR-associated c-Cbl recruits endophilin through an adaptor molecule, CIN-85, thereby facilitating the invagination of plasma membrane [16]. This possible redundant pathway might explain why the inhibition of EGFR ubiquitination by v-Cbl, a dominant-negative form of c-Cbl, is not able to inhibit the internalization of EGFR [17], [18]. Another reason is the absence of a suitable experimental tool by which the detailed status of ubiquitination of surface molecules can be visualized. Although there are a few reports in which the ubiquitination status of surface molecules is monitored by using surface biotinylation, they only demonstrated that ubiquitinated target molecules are present at the plasma membrane [19], [20]. Given that it is necessary to show that non-ubiquitinated target surface molecules undergo ubiquitination immediately before the initiation of internalization, the evidence presented in those studies is not strong enough. Indeed, ubiquitin-harboring model membrane proteins, which are generally employed to analyze ubiquitin-mediated traffic, are able to reach the plasma membrane [21], indicating that some molecules ubiquitinated inside the cells might be transported to the plasma membrane.

In order to overcome the problems mentioned above, we utilized c-MIR, a constitutively active E3 ubiquitin ligase, for B7-2 and MHC II [11]–[13]. We previously showed that transient or stable over-expression of c-MIR induces marked internalization of target surface molecules, and its effect is cancelled by inhibition of target molecule ubiquitination. Based on these findings, we had proposed that c-MIR is a constitutively active E3 ubiquitin ligase and induces the internalization of a surface molecule through ubiquitination of its cytoplasmic tail [11]. c-MIR belongs to a novel family of membrane-bound ubiquitin ligases designated as the MIR/K3 family [14], [22]. All MIR family members possess a variant type of RING (RINGv) domain as an E3 catalytic domain and two transmembrane regions at the amino terminus and the center, respectively. Each MIR family member has specific target molecules: B7-2 and MHC II for c-MIR, and MHC I for MIR1 and 2 [11], [13], [23]–[25]. Previous reports have shown that MIR family members ubiquitinate target molecules through association between the transmembrane regions of E3 and their targets, but it remains unknown how the association is regulated [11], [26]. Our present data showed that T-REx-c-MIR also can be utilized to reveal how c-MIR recognizes the substrate.

To monitor the surface ubiquitination-induced internalization, we employed the exogenously expressed CD8 chimeric molecules as we could not monitor the ubiquitination status of endogenous target molecules (e.g. B7-2) unless the inhibitor for degradation was used in T-REx-c-MIR (Figure S1). Therefore, there is the possibility that the exogenously expressed CD8 chimeric molecules might induce artificial effects (e.g. ER stress) to T-REx-c-MIR, thereby the results obtained from CD8 chimeric molecules-expressing T-REx-c-MIR might not reflect the authentic events observed in the endogenous target molecules (e.g. B7-2, MHC class II). To exclude this possibility, we compared CD8 chimera-expressing T-REx-c-MIR with original T-REx-c-MIR. Both T-REx-c-MIRs showed same phenotypes in terms of down-regulation, ubiquitination status and endocytosis of B7-2 (Figure S2A, B and C). Also, the extent of ER stress was not increased by expression of CD8 chimera (Figure S2D). These results demonstrated that the result obtained in this experiment is not totally artificial one.

Although we previously reported c-MIR as an E3 ubiquitin ligase, c-MIR might have other functions than E3 ubiquitin ligase. Indeed, c-Cbl facilitates internalization of EGFR through binding to CIN-85, and its function does not depend on the E3 ubiquitin ligase activity of c-Cbl [16]. Therefore, we examined the relationship between ubiquitination activity and internalization activity by using several c-MIR mutants. We generated c-MIR mutant whose RINGv domain was mutated (Figure S3A). Also, additional c-MIR mutant whose first hypothetical tyrosine-based motif (Y1) or second hypothetical tyrosine-based motif (Y2) was deleted was generated (Figure S3A). As shown in Figure S3B, C, all c-MIR mutants could not down-regulate and ubiquitinate the target molecules. These results support our idea that c-MIR-mediated internalization depends on the ubiquitin ligase activity of c-MIR in T-REx-c-MIR.

T-REx-c-MIR and FLAG-tagged target molecules present several advantages for the investigation of ubiquitin-mediated membrane trafficking [13], [27]. By combining with the surface biotinylation method, we succeeded for the first time in observing in detail the status of target ubiquitination. Also, the purification method with M2-conjugated matrix is a convenient and excellent means to detect ubiquitinated target molecules. As shown in Figure 3, this method allowed us to conclude that K340 is the dominant site of ubiquitination by c-MIR. In addition, T-REx-c-MIR can be easily modified by retrovirus-mediated gene transfer. We observed that around 80% of the cells were infected with reporter-expressing retrovirus, as judged by the expression of a reporter gene (data not shown). Thus, T-REx-c-MIR can be employed to test whether candidate molecules are indeed involved in ubiquitin-mediated transport.

In this study, we found that c-MIR-mediated internalization was inhibited by chlorpromazine and dansylcadaverine, which are inhibitors of clathrin-dependent internalization. In contrast, nystatin and filipin, which are inhibitors of caveolin-dependent internalization, did not inhibit c-MIR-mediated internalization (data not shown). Also, oligo-ubiquitinated target surface molecules were accumulated by inhibiting internalization (Figure 2C). These results support previous reports demonstrating that the oligo-ubiquitination of plasma membrane proteins induces clathrin-dependent internalization [15], [21].

Our present results give rise to several important points in ubiquitination-mediated internalization. As shown in Figure 3C, the ubiquitination of K364 constitutively took place, but was not a strong internalization signal. In contrast, the ubiquitination of K340 was induced by Dox-mediated c-MIR expression, and was an essential signal for internalization. These results indicated that the ubiquitination of K364 is induced by unidentified endogenous E3 ubiquitin ligases in T-REx-c-MIR. Interestingly, K340 was also demonstrated to be a critical residue for internalization induced by MIR1, viral MIR family member [28]. At present, we cannot explain the difference in the ability to induce internalization between K340 and K364. Nevertheless, we would like to propose that effective internalization requires lysine residues suitably positioned at cytoplasmic tail of the target molecule and a suitable form of ubiquitination chain at critical lysine residues.

## Materials and Methods

### Plasmid construction

Each CD8 chimera was constructed by overlapping PCR as described previously [11]. Substitutions were engineered into CD8 chimera by PCR-based mutagenesis (Promega). Each cDNA of CD8 chimera was subcloned into p3XFLAG-CMV vector (Sigma) to introduce a FLAG epitope tag at the amino terminus. shRNA for UbcH5b/c or luciferase was first subcloned into pcPUR vector [29], and each shRNA cassette was transferred into pSIREN-puro vector (BD). To generate shRNA for UbcH 5b/c, we employed the sequence reported to be able to inhibit both UbcH5b and UbcH5c [15].

### Generation of T-REx-c-MIR

To generate T-REx-c-MIR, BJAB cells were utilized as parent cells. Generation of T-REx-c-MIR cells was performed using the Flp-In ™ T-REx ™ Core Kit (Invitrogen). All procedures were in accordance with the manufacturer's recommendation. His-V5-tagged human c-MIR-expressing cassette, which was used in our previous report [11], was transferred into pcDNA5/FRT/TO vector. Each c-MIR mutant was constructed by overlapping PCR and transferred into pcDNA5/FRT/TO vector [11]. Generated T-REx-c-MIR was transfected with CD8-chimera-expressing plasmids constructed in p3xFLAG-CMV vector, and CD8-chimera-expressing T-REx-c-MIR was selected by incubation with 2 mg/ml G418 (Sigma) for 3–6 weeks.

### Induction of c-MIR in T-REx-c-MIR

T-REx-c-MIR (3×10^5^/ml) was incubated with 1 µg/ml doxycycline (Sigma) in 10% FCS-containing RPMI (Sigma) for indicated times.

### Flow cytometry analysis and antibody

Cells (5×10^5^) were washed with PBS containing 2% fetal calf serum (FCS) and incubated with FITC- or phycoerythrin (PE)-conjugated monoclonal Abs for 30 min at 4°C. After being washed, each sample was fixed with 2% paraformaldehyde solution and flow cytometry analysis was performed with FACSCalibur (BD Biosciences). W6/32 Ab for MHC I, RPA-T8 Ab for CD8, FUN-1 Ab for B7-2, and TÜ36 Ab for MHC II used for FACScan were obtained from BD Biosciences. M2 anti-FLAG Ab (Sigma), F7 anti-HA Ab (Santa Cruz), G-18 anti-His Ab (Santa Cruz), FK2 anti-ubiquitin Ab (AFFINITI Research Products) and anti-V5 Ab (Invitrogen) were used for immunoprecipitation and/or immunoblot analysis.

### Cell surface biotinylation

Cells were incubated with Sulfo-NHS-biotin (2 mg/ml) (Pierce) in PBS (pH 8.0) for 30 min on ice. After incubation, excess biotin was quenched with ice-cold 100 mM glycin. After being chased for the indicated times at 37°C, surface biotinylated cells were used in each experiment.

### Detection of ubiquitinated CD8 chimera

In order to detect the ubiquitination of CD8 chimera, cell pellet was boiled in 1% SDS-containing RIPA buffer (10 mM Tris (pH 7.5), 1% NP40, 0.1% DOC, 0.15 M NaCl, 1 mM EDTA (pH 8.0)), and diluted 10-fold with SDS-free RIPA buffer. After removing cell debris, CD8 chimeric molecules were precipitated with M2 anti-FLAG Ab-coupled sepharose (Sigma). The precipitated CD8 chimeric molecules were eluted with FLAG peptide (150 µg/ml). The eluted sample was subjected to western blot analysis with M2 anti-FLAG Ab or FK2 anti-ubiquitin Ab. To examine whether CD8 chimeric molecules that pre-exists at the plasma membrane before initiation of ubiquitination process by c-MIR are ubiquitinated, surface molecules of CD8-chimera-expressing T-REx-c-MIR cells were biotinylated and followed by incubation with Dox. After being incubated for indicated time, CD8 chimera was purified with M2 anti-FLAG Ab- coupled sepharose as performed above, and further purified with streptavidin-agarose (Pierce), and subjected to western blot analysis with M2 anti-FLAG Ab or FK2 anti-ubiquitin Ab.

### Analysis of surface CD8 chimera stability

Cells were biotinylated as above and cultured in 10% FCS RPMI at 37°C for indicated times. At the end of the chase periods, biotinylated proteins were extracted with 0.1% SDS containing RIPA buffer, purified with streptavidin-agarose, and subjected to western blot with M2 anti-FLAG Ab.

### Internalization assay

Cells were stained with anti-CD8 Ab for 30 min at 4°C. After being washed, cells were cultured for indicated times at 37°C and stained with PE-conjugated goat anti-mouse IgG Ab, followed by FACS analysis at each time point. The absolute value of the surface expression level was determined by subtracting the mean fluorescence intensity (MFI) of cells stained with isotype control Ab from the MFI of cells stained with anti-CD8 Ab. The percentage of CD8 chimera remaining at the cell surface was obtained by dividing the values obtained at each incubation period by the value obtained at time zero. For immunofluorescence microscopy, FITC-labeled CD8 Ab was added to the culture medium of T-REx-c-MIR, and T-REx-c-MIR was cultivated for 10 min at 37 . After being washed, these cells were subjected to analysis with an immunofluorescence microscope.

### Knock-down of E2 by shRNA

Inhibition of E2 ubiquitin conjugating enzymes was performed by retroviral transduction of shRNAs against UbcH5b/c. shRNA-expressing retrovirus was generated by transfecting pSIREN vector that expresses shRNA against UbcH5b/c or control molecule, luciferase, into Phoenix packaging cells. T-REx-c-MIR was infected by spin infection (2,000 rpm for 1 hr at 32°C) with each shRNA-expressing retrovirus. shRNA-expressing T-REx-c-MIR cells were selected by adding puromycin (4 µg/ml).

## Supporting Information

Figure S1 Detection of ubiquitinated B7-2 in T-REx-c-MIR. Original T-REx-c-MIR were incubated with Dox for 8 hr, and cell pellet of incubated original T-REx-c-MIR was boiled in 1% SDS-containing RIPA buffer (10 mM Tris (pH 7.5), 1% NP40, 0.1% DOC, 0.15 M NaCl, 1 mM EDTA (pH 8.0)), and diluted 10-fold with SDS-free RIPA buffer. After removing cell debris, endogenous B7-2 molecule was precipitated with IT 2.2 anti-B7-2 Ab. The precipitated sample was subjected to western blot analysis with BU63 anti-B7-2 Ab or FK2 anti-ubiquitin Ab. Same experiments were performed after degradation of B7-2 was inhibited by adding 2 µM of bafilomycin A1 (Bafi-A1+). In that case, original T-REx-c-MIR was incubated with Dox for 8 hr, and incubated with bafilomycin A1 for the last 5 hr.
(0.20 MB TIF)

Figure S2 Comparison between original T-REx-c-MIR and CD8 chimera-expressing T-REx-c-MIR. (A) Indicated T-REx-c-MIRs were incubated with Dox for 24 hr, and the expression level of surface B7-2, MHC class I (MHC I) and MHC class II (MHC II) was analyzed by FACS. Data from the cells incubated with Dox (open histograms), and the cells incubated without Dox (shaded histograms) are shown. “T-REx-c-MIR” indicates original T-REx-c-MIR. “T-REx-c-MIR-CD8-B7-2-A2” indicates CD8 chimera-expressing T-REx-c-MIR. (B) Original T-REx-c-MIR and CD8 chimera-expressing T-REx-c-MIR were incubated with Dox for 8 hr, and incubated with 2 µM of bafilomycin A1 for the last 5 hr. After incubation, whole cell lysate extracted from each T-REx-c-MIR was incubated with IT 2.2 anti-B7-2 Ab. Precipitated samples were probed with FK-2 anti-ubiquitin Ab (upper) or BU63 anti-B7-2 Ab (lower). The results of original T-REx-c-MIR and CD8 chimera-expressing T-REx-c-MIR were shown in lane 1 and 2 and lane 3 and 4, respectively. (C) Indicated T-REx-c-MIRs were incubated with Dox for 8 hr and cultivated in the presence of FITC-conjugated FUN-1 anti-B7-2 Ab for the last 10 min. Internalized B7-2 was observed with a fluorescence microscope (lower panel). For the quantitative analysis of internalization, surface B7-2 of each T-REx-c-MIR was labeled with FUN-1 anti-B7-2 Ab after being incubated with (+) or without (−) Dox for 8 hr. After cultivation at 37°C for the indicated times, the expression of remaining surface B7-2 was examined by staining with PE-conjugated goat anti-mouse IgG. At each point, the percentage of remaining B7-2 was calculated relative to the value of labeled CD8-B7-2-A2 at 0 min (upper panel). (D) Total RNA from T-REx-c-MIR or CD8 chimera-expressing T-REx-c-MIR either unstimulated (−) or stimulated (+) with Tunicamycin (Tm) (2.5µg/ml) as positive control for 24 h (upper panel) or 12 h (lower panel) was used for RT-PCR analysis. In the upper panel, RT-PCR analysis of XBP1 mRNA splicing was done with a primer set flanking the spliced-out region in XBP1 mRNA. PCR products separated by electrophoresis on 3% agarose gel. In the lower panel, the abundance of GRP78 mRNA was determined by semi-quantitative RT-PCR analysis. PCR products were resolved on 1.5% agarose gel. The results of original T-REx-c-MIR and CD8 chimera-expressing T-REx-c-MIR were shown in lane 1, 3, 5, 7, 9, 11and lane 2, 4, 6, 8, 10, 12, respectively. XBP1(h), hybrid of spliced and unspliced; XBP1(u), unspliced; XBP1(s), spliced; ACTB, beta-actin.
(1.66 MB TIF)

Figure S3 Analysis of c-MIR mutants. (A) Schematic representation of the structure of c-MIR mutants used in this experiment. First putative tyrosine based motif (Y1), second putative tyrosine based motif (Y2), transmembrane domain (TM), and variant RING domain (RINGv) are indicated. (B) Each T-REx-c-MIR expressing indicated c-MIR mutant was incubated with Dox for 24 hr, and the expression level of surface CD8-B7-2-A2 was examined by FACS. (C) Each T-REx-c-MIR was incubated with Dox for 6 hr, and CD8-B7-2-A2 molecules were purified from whole cell lysate as performed in Figure 1D. Purified CD8-B7-2-A2 was analyzed with M2 anti-FLAG and FK2 anti-ubiquitin Abs (upper panel). Bands corresponding to the ubiquitinated CD8-B7-2-A2 is are marked by an asterisk (*) as shown. Also, the expression level of each c-MIR mutant was analyzed by western blot analysis with anti-V5 Ab (lower panel).
(1.60 MB TIF)
