# Supplementary figures and images for: An Excellent Monitoring System for Surface Ubiquitination-Induced Internalization in Mammals
Source: PLoS One. 2008 Jan 30;3(1):e1490. doi: 10.1371/journal.pone.0001490 (PMC2204058; doi:10.1371/journal.pone.0001490)

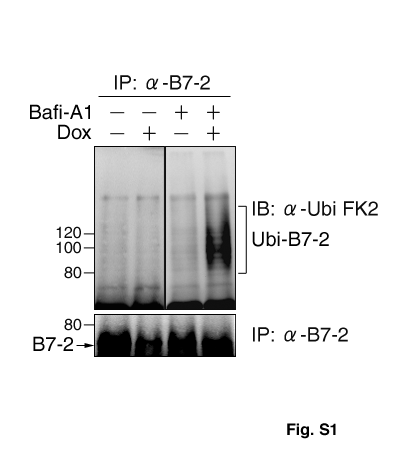

Supplement: Figure S1 — Detection of ubiquitinated B7-2 in T-REx-c-MIR. Original T-REx-c-MIR were incubated with Dox for 8 hr, and cell pellet of incubated original T-REx-c-MIR was boiled in 1% SDS-containing RIPA buffer (10 mM Tris (pH 7.5), 1% NP40, 0.1% DOC, 0.15 M NaCl, 1 mM EDTA (pH 8.0)), and diluted 10-fold with SDS-free RIPA buffer. After removing cell debris, endogenous B7-2 molecule was precipitated with IT 2.2 anti-B7-2 Ab. The precipitated sample was subjected to western blot analysis with BU63 anti-B7-2 Ab or FK2 anti-ubiquitin Ab. Same experiments were performed after degradation of B7-2 was inhibited by adding 2 µM of bafilomycin A1 (Bafi-A1+). In that case, original T-REx-c-MIR was incubated with Dox for 8 hr, and incubated with bafilomycin A1 for the last 5 hr. (0.20 MB TIF) [file pone.0001490.s001.tif]

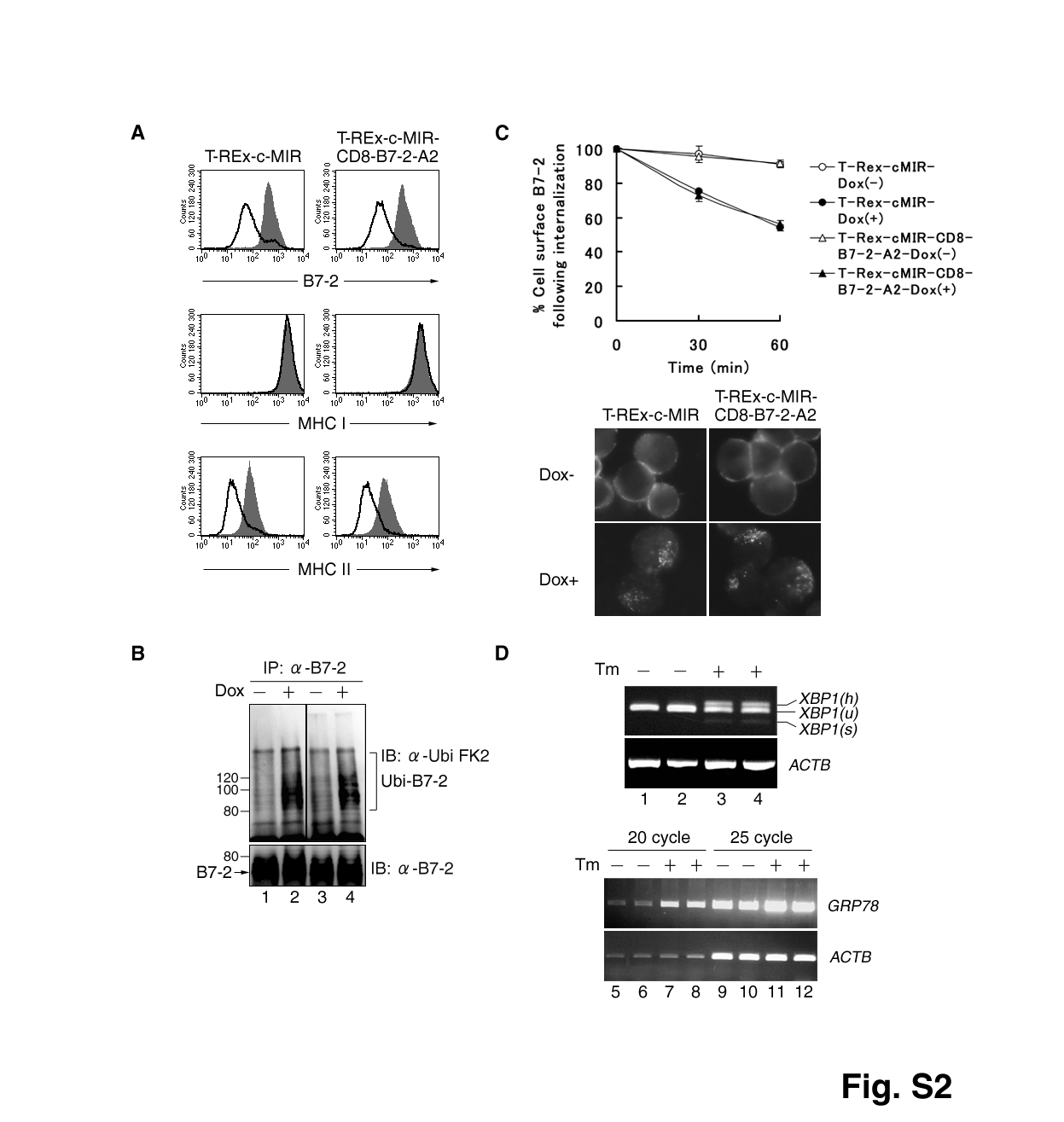

Supplement: Figure S2 — Comparison between original T-REx-c-MIR and CD8 chimera-expressing T-REx-c-MIR. (A) Indicated T-REx-c-MIRs were incubated with Dox for 24 hr, and the expression level of surface B7-2, MHC class I (MHC I) and MHC class II (MHC II) was analyzed by FACS. Data from the cells incubated with Dox (open histograms), and the cells incubated without Dox (shaded histograms) are shown. “T-REx-c-MIR” indicates original T-REx-c-MIR. “T-REx-c-MIR-CD8-B7-2-A2” indicates CD8 chimera-expressing T-REx-c-MIR. (B) Original T-REx-c-MIR and CD8 chimera-expressing T-REx-c-MIR were incubated with Dox for 8 hr, and incubated with 2 µM of bafilomycin A1 for the last 5 hr. After incubation, whole cell lysate extracted from each T-REx-c-MIR was incubated with IT 2.2 anti-B7-2 Ab. Precipitated samples were probed with FK-2 anti-ubiquitin Ab (upper) or BU63 anti-B7-2 Ab (lower). The results of original T-REx-c-MIR and CD8 chimera-expressing T-REx-c-MIR were shown in lane 1 and 2 and lane 3 and 4, respectively. (C) Indicated T-REx-c-MIRs were incubated with Dox for 8 hr and cultivated in the presence of FITC-conjugated FUN-1 anti-B7-2 Ab for the last 10 min. Internalized B7-2 was observed with a fluorescence microscope (lower panel). For the quantitative analysis of internalization, surface B7-2 of each T-REx-c-MIR was labeled with FUN-1 anti-B7-2 Ab after being incubated with (+) or without (−) Dox for 8 hr. After cultivation at 37°C for the indicated times, the expression of remaining surface B7-2 was examined by staining with PE-conjugated goat anti-mouse IgG. At each point, the percentage of remaining B7-2 was calculated relative to the value of labeled CD8-B7-2-A2 at 0 min (upper panel). (D) Total RNA from T-REx-c-MIR or CD8 chimera-expressing T-REx-c-MIR either unstimulated (−) or stimulated (+) with Tunicamycin (Tm) (2.5µg/ml) as positive control for 24 h (upper panel) or 12 h (lower panel) was used for RT-PCR analysis. In the upper panel, RT-PCR analysis of XBP1 mRNA splicing was done with [file pone.0001490.s002.tif]

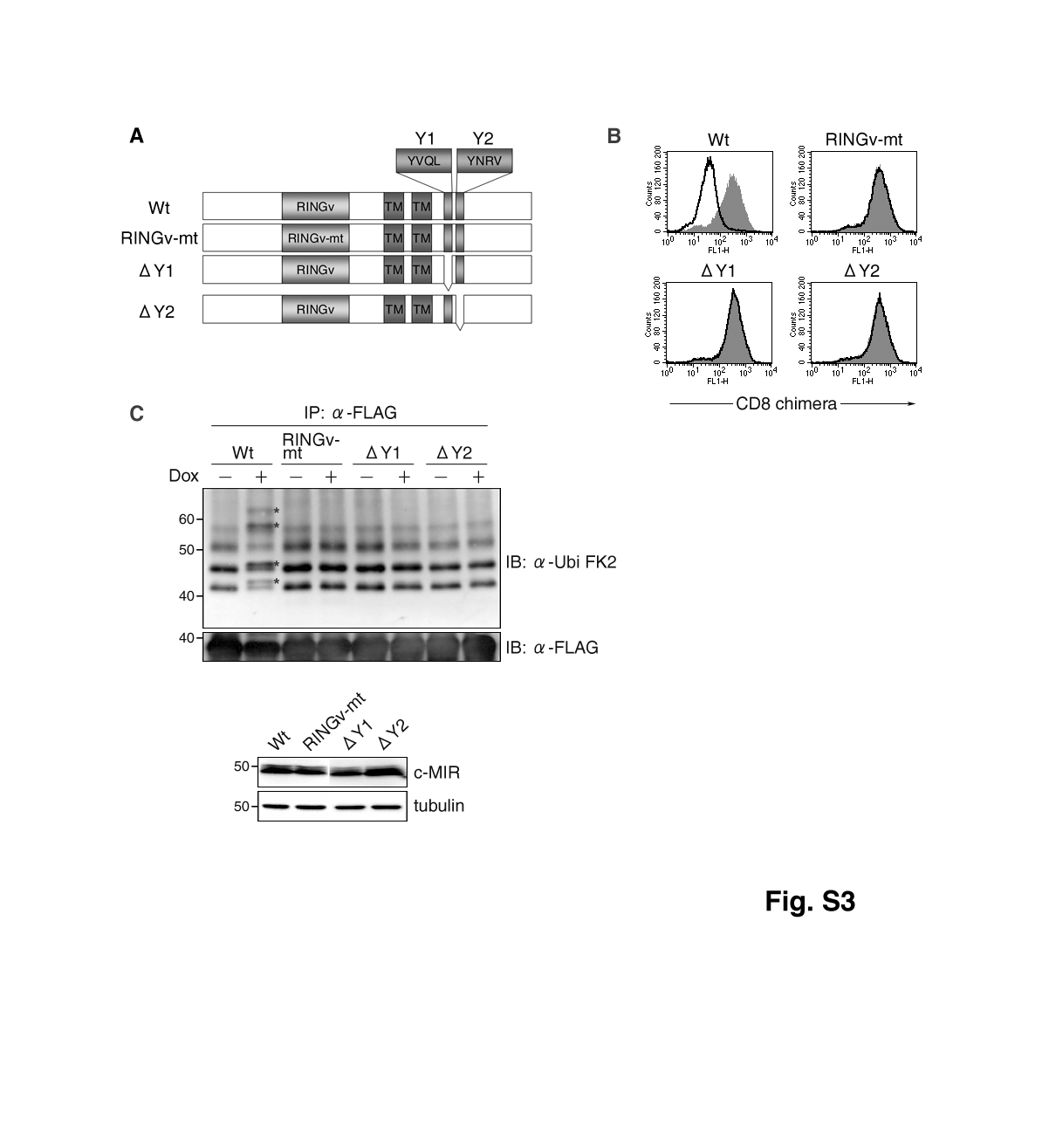

Supplement: Figure S3 — Analysis of c-MIR mutants. (A) Schematic representation of the structure of c-MIR mutants used in this experiment. First putative tyrosine based motif (Y1), second putative tyrosine based motif (Y2), transmembrane domain (TM), and variant RING domain (RINGv) are indicated. (B) Each T-REx-c-MIR expressing indicated c-MIR mutant was incubated with Dox for 24 hr, and the expression level of surface CD8-B7-2-A2 was examined by FACS. (C) Each T-REx-c-MIR was incubated with Dox for 6 hr, and CD8-B7-2-A2 molecules were purified from whole cell lysate as performed in Figure 1D. Purified CD8-B7-2-A2 was analyzed with M2 anti-FLAG and FK2 anti-ubiquitin Abs (upper panel). Bands corresponding to the ubiquitinated CD8-B7-2-A2 is are marked by an asterisk (*) as shown. Also, the expression level of each c-MIR mutant was analyzed by western blot analysis with anti-V5 Ab (lower panel). (1.60 MB TIF) [file pone.0001490.s003.tif]
